# Duodenal neuroendocrine tumor successfully removed by endoscopic submucosal dissection with adaptative traction device

**DOI:** 10.1055/a-2291-9448

**Published:** 2024-04-09

**Authors:** Elena De Cristofaro, Jérôme Rivory, Thomas Walter, Jérémie Jacques, Timothée Wallenhorst, Pierre Lafeuille, Mathieu Pioche

**Affiliations:** 1 Gastroenterology Unit, Department of Systems Medicine, University of Rome Tor Vergata, Rome, Italy; 2 Gastroenterology and Endoscopy Unit, Edouard Herriot Hospital, Hospices Civils de Lyon, Lyon, France; 3 Department of Medical Oncology, Edouard Herriot Hospital, Hospices Civils de Lyon, Lyon, France; 4 Gastroenterology and Endoscopy Unit, Dupuytren University Hospital, Limoges, France; 5 Gastroenterology and Endoscopy Unit, Pontchaillou University Hospital, Rennes, France

Duodenal neuroendocrine tumors (D-NETs) are uncommon neoplastic entities 1 . The current guidelines advocate for resection when the lesion is ≤20 mm in size, in the absence of lymph node involvement 2 . However, the utility of endoscopic resection remains unclear, especially due to the risk of incomplete resection 3 . Endoscopic submucosal dissection (ESD) is an attractive technique because it offers the potential to achieve en bloc resection with clear margins. In a previous retrospective study, ESD for D-NETs achieved a 100% en bloc resection rate; however, the R0 resection rate is far from perfect and the perforation rate is not null 4 . The use of a traction device, with double clips and rubber band traction, has been described previously to improve the exposure of the tiny submucosal space 5 .

In this case, we describe the benefits of using an adaptative traction device (A-TRACT; Hospices Civils de Lyon, Lyon, France) during duodenal ESD ( Video 1 ).

Use of an adaptative traction device with endoscopic submucosal dissection for successful removal of a duodenal neuroendocrine tumor.Video 1

A 76-year-old patient was referred for removal of a D-NET of 8 mm in size, located in the upper part of the duodenal bulb, just behind the pylorus. An ESD was indicated to ensure R0 resection. After circumferential incision and trimming, the two loops of A-TRACT 2 were fixed by two clips to the lesion edges and another clip was used to affix the rubber band to the opposite mucosal wall, allowing exposure of the lesion previously hidden by the pylorus ( Fig. 1 ). The dissection was started with good traction, allowing the cut line to be clearly identified. After half of the lesion had been cut, traction was tightened to optimize visualization, and the dissection was safely completed in 40 minutes without adverse events. The histopathology revealed a G1 NET with lateral free margins but without deep free margins.

**Fig. 1:**
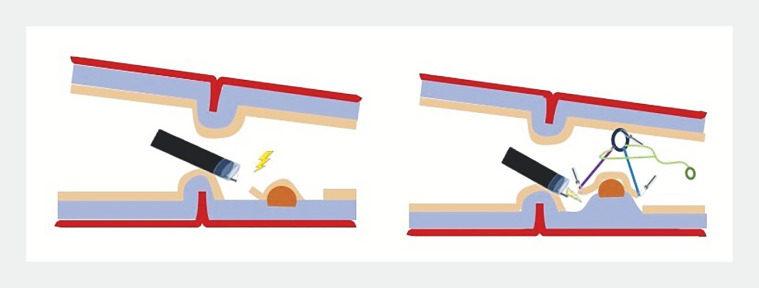
Schematic representation of the adaptive traction device (A-TRACT 2; Hospices Civils de Lyon, Lyon, France), allowing visualization of the lesion, which was previously hidden by the pylorus, and optimal exposure of the submucosa.

We hypothesize that this traction device could facilitate duodenal ESD, which is known to be technically challenging and with high risk of adverse events.

Endoscopy_UCTN_Code_TTT_1AO_2AG_3AD

## Notes

Endoscopy E-Videos https://eref.thieme.de/e-videos
*E-Videos* is an open access online section of the journal *Endoscopy* , reporting on interesting cases and new techniques in gastroenterological endoscopy. All papers include a high-quality video and are published with a Creative Commons CC-BY license. Endoscopy E-Videos qualify for HINARI discounts and waivers and eligibility is automatically checked during the submission process. We grant 100% waivers to articles whose corresponding authors are based in Group A countries and 50% waivers to those who are based in Group B countries as classified by Research4Life (see: https://www.research4life.org/access/eligibility/ ). This section has its own submission website at https://mc.manuscriptcentral.com/e-videos .
